# Supplementary material for: Characterization of Biological Pathways Regulating Acute Cold Resistance of Zebrafish
Source: Int J Mol Sci. 2021 Mar 16;22(6):3028. doi: 10.3390/ijms22063028 (PMC8001686; doi:10.3390/ijms22063028)
Supplement: Supplementary file 1 [file ijms-22-03028-s001.zip › Supplemental Materials/Supplemental Figures.docx]

**SUPPLEMENTAL FIGURES**

**
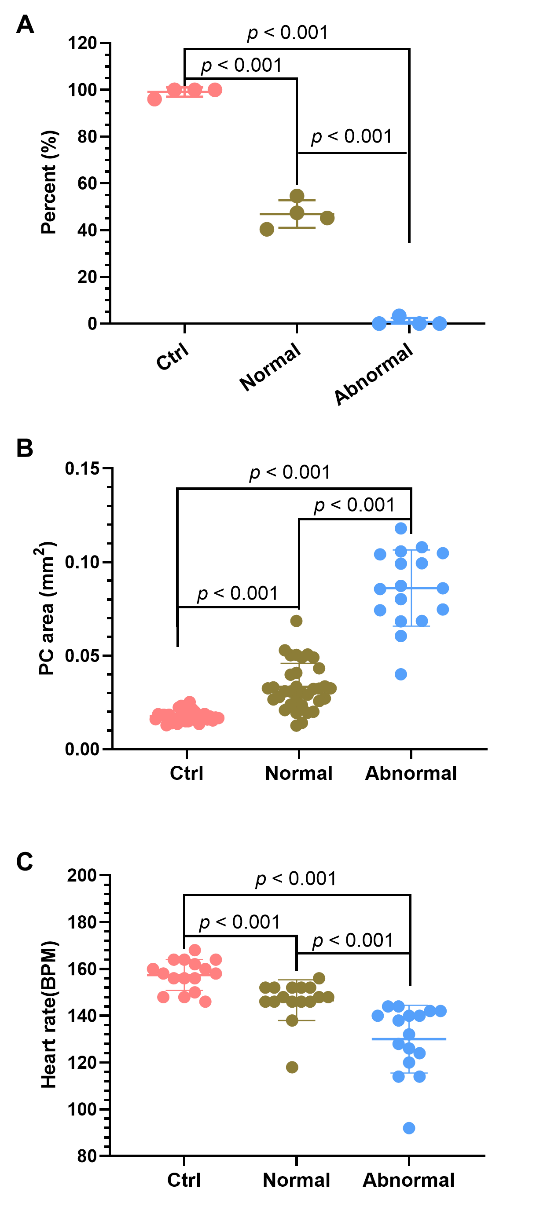
**

**Figure S1.** **Morphological parameters affected by lethal cold exposure.** (**A**) Percent of fish with deflated swim bladder. The bars indicate averages of 3 independent experiments and 20 - 50 larvae for each group were checked in the experiments. The error bars represent standard deviations. (**B**) Area of pericardial cavity (PC). Sample numbers were 34, 34 and 17. (**C**) Heart rate (beat per minute, BPM; n = 16 for all the groups). Zebrafish larvae at 96 hpf were exposed to 10 ^o^C for 24 h and rewarmed at 28 ^o^C for another 24 h. Ctrl: time-match controls not exposed to cold stress, normal: the fish with overall normal morphologies, abnormal: the fish demonstrated obvious morphological defects and could not swim.


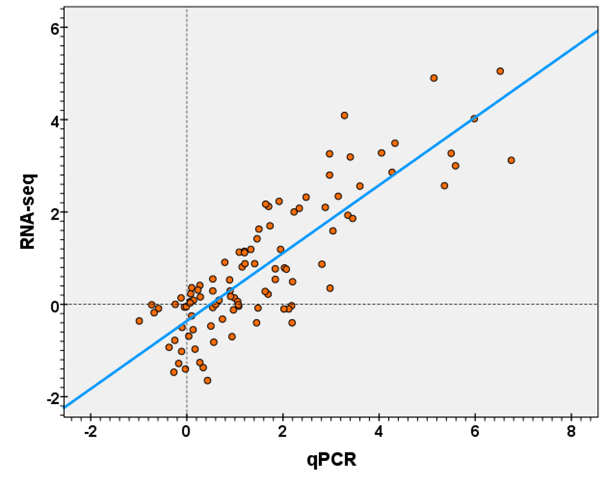


**Figure S2. Correlation between the RNA-seq and qPCR results.** The log2Foldchange of gene expression detected by RNA-seq and qPCR were analyzed by SPSS to calculate the Pearson correlation coefficient.


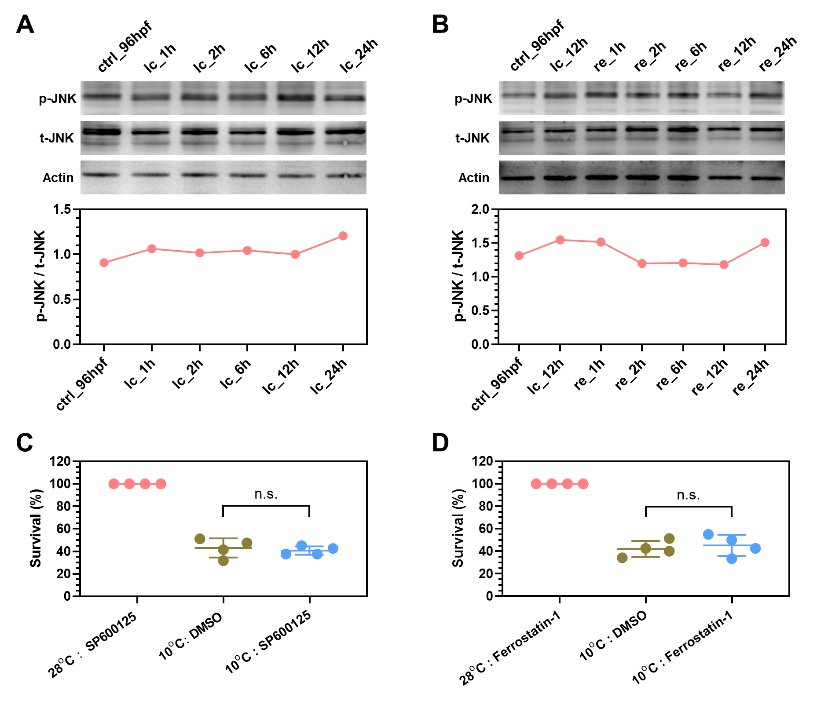


**Figure S3. JNK and ferroptosis were not involved in regulating cold resistance of zebrafish larvae.** (**A and B**) Phosphorylation level of JNK didn’t change during cold stress (A) and recovery at normal temperature (B). The line charts below the western blots indicate ratio of the phosphorylated to the total protein of JNK. (**C**) Treatment with the JNK inhibitor SP600125 (10 μM) and (**D**) The ferroptosis inhibitor ferrostatin-1 (10 μM) had no effect on cold resistance.

**
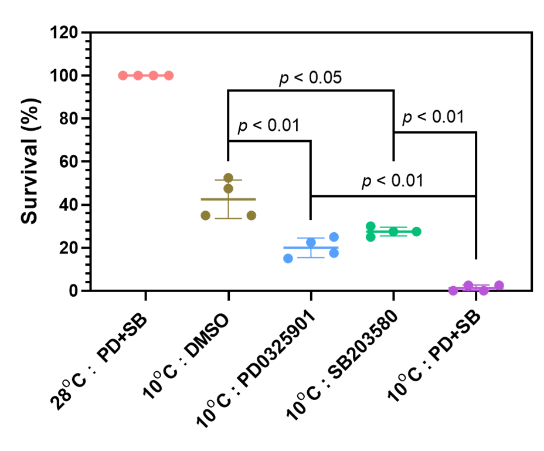
**

**Figure S4. Simultaneous inhibition of ERK and p38 MAPKs sensitized zebrafish larvae to cold stress.** Zebrafish larvae were exposed to 10 ^o^C for 24 h in the presence of PD0325901 (5 μM), SB203580 (50 μM) or both. After cold treatment, the old medium was changed with fresh medium preconditioned at 10 ^o^C. The fish were returned to 28 ^o^C and incubated for another 24 h. The dead fish were removed and recorded occasionally during recovery.

**
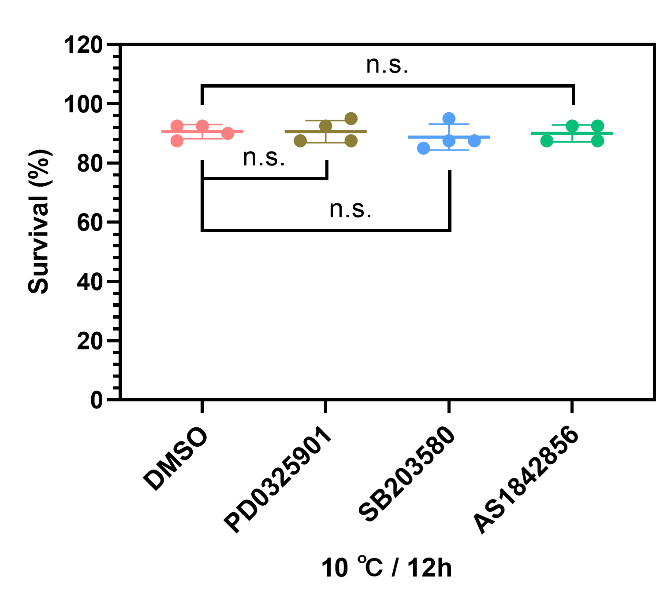
**

**Figure S5. Treatment with inhibitors of Foxo1, ERK and p38 demonstrated no effects on survival upon exposure to cold stress for 12 h.** Zebrafish larvae were exposed to 10 ^o^C for 12 h in the presence of AS1842856 (0.5 μM) PD0325901 (5 μM) or SB203580 (50 μM).


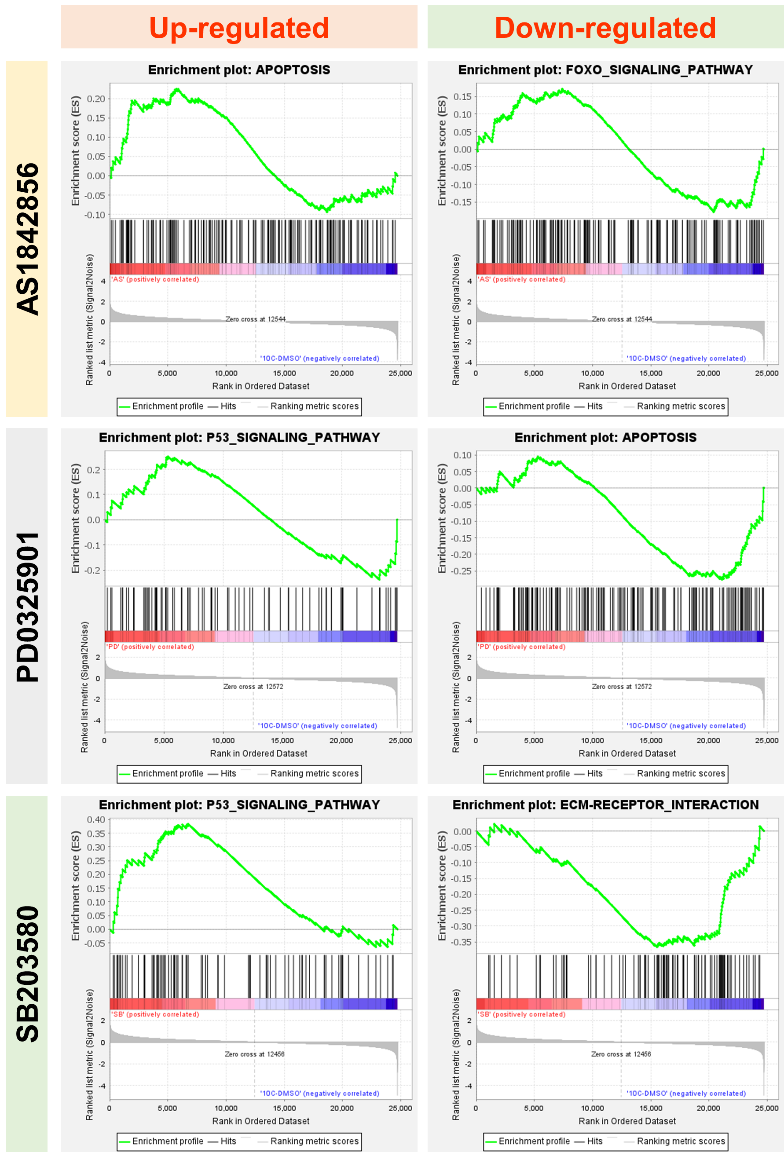


**Figure S6. Representative KEGG pathways affected by AS1842856, PD0325901 and SB203580 upon lethal cold stress.** The whole gene expression datasets of samples for 10^o^C_AS, 10^o^C_PD and 10^o^C_SB were compared with those of 10^o^C_DMSO through GSEA (gene set enrichment analysis) to identify significantly up- and down-regulated KEGG pathways.


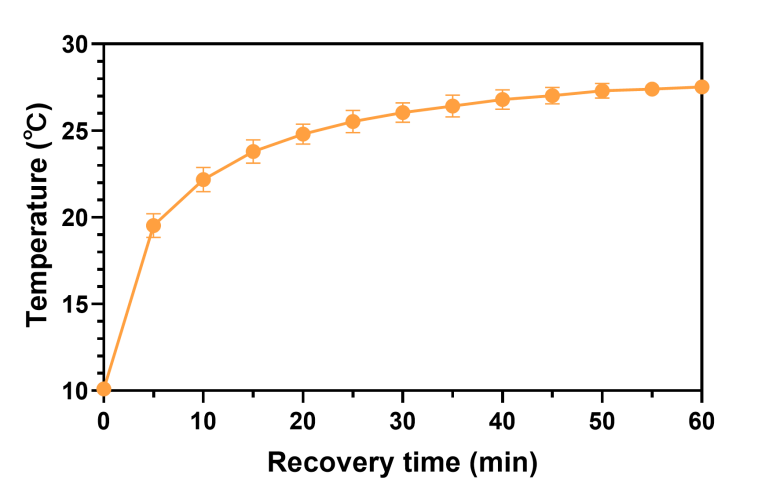


**Figure S7. Increase of water temperature during rewarming.** The plates with 8 mL cold medium were transferred from 10 ^o^C to 28 ^o^C. Temperature of the medium was measured using an electron thermometer at 5 min intervals.
